# Assessment of healthcare worker preparedness and health literacy for Marburg virus disease in Nigeria: A cross-sectional study

**DOI:** 10.4102/sajid.v41i1.764

**Published:** 2026-01-28

**Authors:** Emmanuel O. Oisakede, Daniel Asogun, Osahon Otaigbe, Iziengbe Iyoriobhe, Emmanuel O. Erhieyovwe, Airenakho Emorinken, Martin Nwosu, Uyi M. Osamudiamen, David Olawade

**Affiliations:** 1 Department of Clinical Oncology, Leeds Teaching Hospitals Trust, Leeds, United Kingdom; 2 Department of Health Research, University of Leeds, Leeds, United Kingdom; 3 Department of Internal Medicine, Edo Specialist Hospital, Benin City, Nigeria; 4 Department of Community Medicine, Irrua Specialist Teaching Hospital, Irrua, Nigeria; 5 Department of Internal Medicine, Irrua Specialist Teaching Hospital, Irrua, Nigeria; 6 Department of Research and Innovation, Manchester University NHS Foundation Trust, Manchester, United Kingdom; 7 Division of Rheumatology, Department of Internal Medicine, Irrua Specialist Teaching Hospital, Irrua, Nigeria; 8 Department of Medicine, Ambrose Alli University, Ekpoma, Nigeria; 9 Department of Public Health, University of Chester, Chester, United Kingdom; 10 Department of Allied and Public Health, School of Health, Sport and Bioscience, University of East London, London, United Kingdom; 11 Department of Research and Innovation, Medway NHS Foundation Trust, Gillingham, United Kingdom; 12 Department of Public Health, York St John University, London, United Kingdom

**Keywords:** health literacy, Marburg virus disease, preparedness, healthcare workers, Nigeria

## Abstract

**Background:**

Marburg virus disease (MVD) poses an emerging threat to Nigeria, particularly following the 2022 outbreak in neighbouring Ghana. With Nigeria already managing Lassa fever and Mpox outbreaks, assessing healthcare workers’ preparedness at viral haemorrhagic disease reference centres is crucial for effective outbreak response.

**Objectives:**

This study aimed to assess healthcare workers’ knowledge, attitudes and preparedness regarding MVD at Nigeria’s primary viral haemorrhagic fever reference centre.

**Method:**

A cross-sectional study was conducted at Irrua Specialist Teaching Hospital, from May 2024 to October 2024. Healthcare workers were recruited using simple random sampling and data collected via semi-structured questionnaires. Descriptive and inferential statistics were analysed using Stata 17.

**Results:**

Of the 216 participants, 126 (58.3%) were doctors and 90 (41.7%) were nurses. Doctors demonstrated significantly higher knowledge of MVD symptoms (65.9% vs 46.7%, *p* < 0.001) and risk factors, with fever being the most recognised symptom (68.0%). Only 19.1% of doctors and 10.0% of nurses had received formal MVD training. Confidence in hospital preparedness was paradoxically lower among doctors (32.5%) than nurses (65.6%, *p* < 0.001). Most participants felt inadequately equipped with personal protective equipment, with only 38.1% of doctors and 48.9% of nurses reporting adequate protection.

**Conclusion:**

Significant gaps exist in MVD health literacy and outbreak preparedness among Nigerian healthcare workers at a major viral haemorrhagic disease centre.

**Contribution:**

Enhanced training programmes, improved resource allocation and systematic preparedness protocols are urgently needed to strengthen Nigeria’s capacity for MVD outbreak response.

## Introduction

Marburg virus disease (MVD), previously known as Marburg haemorrhagic fever, is a severe viral haemorrhagic fever caused by Marburg virus (MARV) of the Filoviridae family, with case fatality rates approaching 88%.^1,2^ Since its discovery in 1967 following simultaneous outbreaks in Marburg and Frankfurt in Germany and Belgrade in Serbia, MARV has caused 16 documented outbreaks globally, with all originating in Africa.^3,4^ Recent outbreaks in Equatorial Guinea and Tanzania in 2023 underscore the continuing threat this pathogen poses to African healthcare systems.^3^

The virus comprises two distinct species: MARV and Ravn virus, which exhibit approximately 20% genetic divergence but cause clinically indistinguishable disease.^4,5^ The MARV transmission occurs zoonotically from Egyptian fruit bats (*Rousettus aegyptiacus*) and other bat species or through human-to-human contact with infected blood, secretions and bodily fluids.^4^ The clinical presentation begins with non-specific symptoms, including fever, myalgia and headache, progressing to severe gastrointestinal symptoms and potentially fatal haemorrhagic manifestations.^6,7^ Diagnosis relies on specialised laboratory techniques, including reverse transcription polymerase chain reaction (RT-PCR), enzyme-linked immunosorbent assay (ELISA) and virus isolation although these may be unavailable in resource-limited settings where outbreaks typically occur.^8,9^ Currently, no licensed vaccines or specific antiviral treatments exist, making supportive care the cornerstone of management.^6,10^

Nigeria, despite having no recorded MVD cases, faces increasing risk following Ghana’s 2022 outbreak, the first in West Africa and geographically closest to Nigeria’s borders.^3^ This threat is particularly concerning given Nigeria’s existing burden of viral haemorrhagic diseases, including endemic Lassa fever and recent Mpox outbreaks. Healthcare worker preparedness at viral haemorrhagic disease reference centres represents a critical component of outbreak response capability, as these facilities would serve as frontline treatment centres during any MVD emergency. Understanding the relationship between health literacy and outbreak preparedness is essential for strengthening healthcare system resilience and developing targeted interventions.^11^

This study therefore assessed healthcare workers’ knowledge, attitudes and preparedness regarding MVD at Nigeria’s primary viral haemorrhagic disease reference centre, with the specific objectives of determining healthcare workers’ knowledge of MVD symptoms and transmission, evaluating their attitudes and perceptions towards the disease and assessing their level of preparedness for potential outbreaks. We addressed three key questions: What do healthcare workers know about MVD? What are their attitudes and perceptions towards MVD? How prepared are they for a potential MVD outbreak?

## Research methods and design

### Study design and setting

This descriptive cross-sectional study employed semi-structured questionnaires administered electronically and followed the ‘Strengthening the Reporting of Observational Studies in Epidemiology’ (STROBE) guidelines.^12^ Data collection occurred over five months from May 2024 to October 2024 at Irrua Specialist Teaching Hospital (ISTH), a tertiary centre in Southern Nigeria. The ISTH houses an Institute for Viral Hemorrhagic fevers and is Nigeria’s primary regional reference centre for viral haemorrhagic diseases.^13^ The ISTH serves Edo State and neighbouring Delta, Kogi and Ondo States, receiving patients nationwide because of its specialised viral haemorrhagic fever capabilities. The facility is also a training centre for medical and nursing students from multiple institutions.^14^

### Population and sampling

The study population comprised medical doctors and nurses working at ISTH. Given the absence of published staffing data, sample size calculation used estimates from similar Nigerian tertiary institutions (70 doctors, 220 nurses).^15^ Using Epi Info STATCALC with 95% confidence interval, the minimum required sample was 168 participants.^16^ Simple random sampling was employed to recruit 216 healthcare workers who provided informed consent.

### Data collection and instrumentation

Self-administered structured questionnaires captured sociodemographic characteristics, MVD knowledge, attitudes, perceptions and preparedness measures. The instrument was adapted from validated frameworks used in previous infectious disease studies^17,18^ and pretested among 20 healthcare workers (excluded from final analysis) to ensure clarity and reliability. Questionnaires were administered via Google Forms or paper format based on participant preference.

### Data analysis

Data analysis was conducted using Stata 17. Descriptive statistics summarised categorical variables using frequencies and proportions, while chi-square tests identified significant associations between variables. Results are presented as absolute numbers and percentages, with statistical significance set at *p* < 0.05.

### Ethical considerations

Ethical approval was obtained from the Health Research Ethics Committee of Irrua Specialist Teaching Hospital on 20 May 2024. The ethical registration number is: NHREC/29/03/2017 and the protocol number is: ISTH/HREC/20240904/591. Participants provided written informed consent before questionnaire completion, with confidentiality maintained through secure data storage on password-protected devices accessible only to researchers.

## Results

### Participants’ characteristics

Of the 216 participants, 126 (58.3%) were doctors and 90 (41.7%) were nurses. Nurses had a significantly higher median age (38 years) than doctors (29 years, *p* < 0.001). Most doctors (69.1%) were in early career stages with 0–5 years of experience, while 43.3% of nurses had over 10 years of experience. Significant gender differences existed, with 75.6% of nurses being female compared to 30.2% of doctors (*p* < 0.001). Participants were distributed across various departments, with the largest representations in Obstetrics and Gynaecology (24.6% of doctors, 12.2% of nurses) and Surgery (19.1% of doctors, 14.4% of nurses). The distribution of the doctors’ and nurses’ departments is shown in Table 1.

**TABLE 1:** Sociodemographic characteristics of respondents.

Variable	Doctors *N* = 126			Nurses *N* = 90			χ^2^	*p*-value
	*n*	%	Median age	*n*	%	Median age		
**Age group (years)**								
16–24	7	5.6	-	10	11.1	-	29.365	< 0.001
25–33	72	57.1	-	23	25.6	-	-	-
34–42	35	27.8	-	27	30.0	-	-	-
43–51	12	9.5	-	30	33.3	-	-	-
	-	-	29 (29, 38)	-	-	38 (29, 47)	-	< 0.001
**Sex**								
Female	38	30.2	-	68	75.6	-	63.659	< 0.001
Male	81	64.3	-	9	10.0	-	-	-
Prefer not to say	7	5.6	-	13	14.4	-	-	-
**Years of experience**								
0–5	87	69.1	-	33	36.7	-	22.418	< 0.001
6–10	11	8.7	-	18	20.0	-	-	-
> 10	28	22.2	-	39	43.3	-	-	-
**Department**								
Anaesthesiology	2	1.6	-	3	3.3	-	15.617	0.075
Community medicine	5	4.0	-	9	10.0	-	-	-
Dentistry	1	0.8	-	5	5.6	-	-	-
Internal medicine	22	17.5	-	12	13.3	-	-	-
Mental health	5	4.0	-	2	2.2	-	-	-
Obstetrics and gynaecology	31	24.6	-	11	12.2	-	-	-
Paediatrics	9	7.1	-	10	11.1	-	-	-
Radiology	6	4.8	-	6	6.7	-	-	-
Surgery	24	19.1	-	13	14.4	-	-	-
Others	21	16.7	-	19	21.1	-	-	-

Note: Some percentages may not sum up to 100 because of rounding.

### Knowledge of Marburg virus disease

**FIGURE 1:**
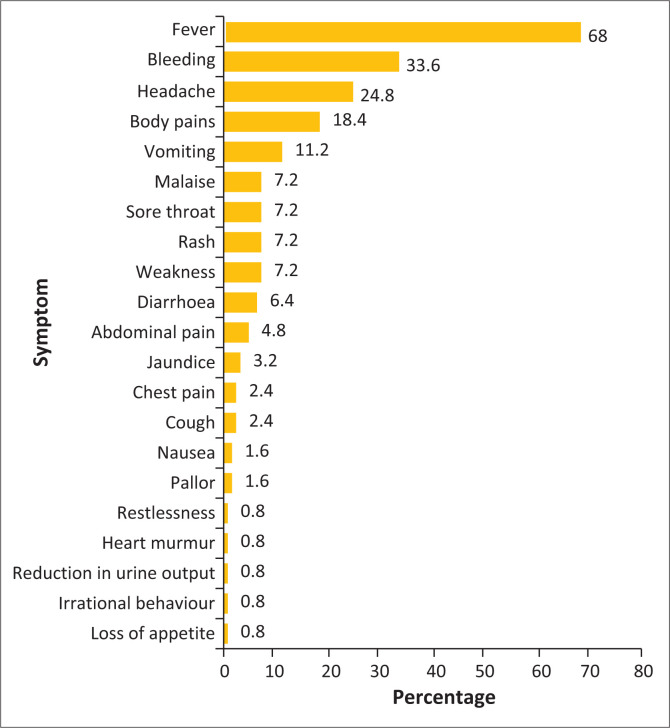
Respondents’ awareness of symptoms associated with Marburg virus disease.

Overall, as shown in Figure 1, 125 participants (57.9%) demonstrated awareness of MVD symptoms, with doctors showing significantly higher knowledge levels (65.9%) than nurses (46.7%). Fever was the most recognised symptom (68.0%), followed by bleeding (33.6%), headache (24.8%), body pains (18.4%) and vomiting (11.2%). As highlighted in Table 2, doctors demonstrated superior recognition of fever (77.1% vs. 50.0%, *p* < 0.001) and bleeding (44.6% vs. 11.9%) as symptoms, while nurses more frequently identified vomiting (16.7% vs. 8.4%).

**TABLE 2:** Awareness of symptoms associated with Marburg virus disease.

Variable	Doctors *N* = 83		Nurses *N* = 42		χ^2^	*p*-value
	*n*	%	*n*	%		
Fever	64	77.1	21	50.0	16.588	< 0.001
Bleeding	37	44.6	5	11.9	-	-
Headache	19	22.9	12	28.6	-	-
Body pains	19	22.9	4	9.5	-	-
Vomiting	7	8.4	7	16.7	-	-

Formal MVD training was limited, with only 19.1% of doctors and 10.0% of nurses having received specific training. Healthcare workers obtained information primarily through online resources (54.8% of doctors, 42.2% of nurses), workshops or seminars (33.3% of doctors, 48.9% of nurses) and professional journals (42.9% of doctors, 32.2% of nurses).

Knowledge of transmission risk factors varied significantly between professional groups (see Table 3). Doctors demonstrated superior understanding of key risk factors including close contact with infected individuals (69.1% vs. 46.7%, *p* = 0.001), contact with infected fluids or surfaces (61.1% vs. 32.2%, *p* < 0.001), prolonged bat exposure (40.5% vs. 11.1%, *p* < 0.001) and participation in burial ceremonies (35.7% vs. 10.0%, *p* < 0.001).

**TABLE 3:** Respondents’ knowledge sources of Marburg virus disease.

Variable	Doctors *N* = 126		Nurses *N* = 90		χ^2^	*p*-value
	*n*	%	*n*	%		
**Received formal training on MVD**						
Yes	24	19.1	9	10.0	3.320	0.068
No	102 (80.9)	-	81	90.0	-	-
Source of information about infectious diseases*						
Online resources	69	54.8	38	42.2	3.302	0.069
Workshops or seminars	42	33.3	44	48.9	5.302	0.021
Professional journals	54	42.9	29	32.2	2.510	0.113
Official health bulletins	31	24.6	18	20.0	0.634	0.426
Knowledge of risk factors*						
Close contact with infected individuals	87	69.1	42	46.7	10.932	0.001
Contact with infected fluid, secretions or surfaces	77	61.1	29	32.2	17.532	< 0.001
Needle stick injuries	43	34.1	32	35.6	0.047	0.828
Prolonged exposure to bats	51	40.5	10	11.1	22.339	< 0.001
Burial ceremonies involving close contact with the dead body	45	35.7	9	10.0	18.514	< 0.001
Eating contaminated food	27	21.4	18	20.0	0.065	0.799
Sexual contact with an infected partner	30	23.8	15	16.7	1.624	0.203

* Multiple response.

### Attitudes, perceptions and preparedness

As detailed in Table 4, confidence levels varied markedly between doctors and nurses. Regarding hospital preparedness, nurses expressed significantly higher confidence (65.6%) compared to doctors (32.5%, *p* < 0.001). Similarly, more nurses felt confident in their ability to educate patients and communities about MVD (60.0% vs 42.9%, *p* = 0.009).

**TABLE 4:** Respondents’ attitudes, perceptions and preparedness towards an outbreak of Marburg virus disease.

Variable	Doctors *N* = 126		Nurses *N* = 90		χ^2^	*p*-value
	*n*	%	*n*	%		
**Confidence in knowledge of MVD**						
Confident	36	28.6	28	31.1	0.974	0.614
Neutral	40	31.8	23	25.6	-	-
Not confident	50	39.7	39	43.3	-	-
**Confidence in the ability to educate patients and the community about MVD**						
Confident	54	42.9	54	60.0	9.495	0.009
Neutral	42	33.3	14	15.6	-	-
Not confident	30	23.8	22	24.4	-	-
**Confidence in hospitals preparedness to handle MVD cases**						
Confident	41	32.5	59	65.6	23.428	< 0.001
Neutral	42	33.3	13	14.4	-	-
Not confident	43	34.1	18	20.0	-	-
**Believes there are established protocols in hospital for handling suspected cases of MVD**						
Yes	46	36.5	52	57.8	9.583	0.002
No	80	63.5	38	42.2	-	-
**Feels adequately equipped with protective gear to handle potential cases of MVD**						
Yes	48	38.1	44	48.9	22.527	< 0.001
No	55	43.7	13	14.4	-	-
Partially	23	18.2	33	36.7	-	-
**Perceived risk of MVD outbreak in hospital**						
It may happen	75	59.5	51	56.7	1.503	0.472
It is unlikely to happen	48	38.1	34	37.8	-	-
It is impossible	3	2.4	5	5.6	-	-
**Believes health care workers are at higher risk of contracting MVD compared to the general population**						
Yes	102	81.0	56	62.2	9.542	0.008
No	9	7.1	11	12.2	-	-
Maybe	15	11.9	23	25.6	-	-
Measures to enhance awareness and preparedness for MVD among health workers*						
Training of health workers	107	84.9	60	66.7	9.974	**0.002**
Regular seminars	87	69.1	42	46.7	10.932	**0.001**
Sharing of hand flyers and educational materials	77	61.1	39	43.3	6.674	0.010

MVD, Marburg virus disease.

* Multiple response some percentages may not sum up to 100 because of rounding.

Personal protective equipment adequacy was a major concern, with only 38.1% of doctors and 48.9% of nurses feeling adequately equipped. Most participants (63.5% of doctors, 42.2% of nurses) believed their hospital lacked established protocols for managing suspected MVD cases.

Risk perception showed that 81.0% of doctors considered healthcare workers at higher risk than the general population, compared to 62.2% of nurses (*p* = 0.008). Most participants viewed an MVD outbreak as possible (59.5% of doctors, 56.7% of nurses).

To enhance preparedness, participants recommended training programmes (84.9% of doctors, 66.7% of nurses), regular seminars (69.1% of doctors, 46.7% of nurses) and educational materials distribution (61.1% of doctors, 43.3% of nurses).

## Discussion

This study reveals significant gaps in MVD health literacy and preparedness among healthcare workers at Nigeria’s primary viral haemorrhagic disease reference centre. The findings demonstrate professional disparities in knowledge, concerning deficits in formal training and inadequate preparedness infrastructure.

The superior MVD knowledge among doctors compared to nurses reflects differences in clinical training and specialised exposure to complex infectious diseases. However, the overall knowledge levels remain suboptimal, with only 57.9% of participants demonstrating symptom awareness. This finding aligns with a Guinea-based study where only 35.3% of healthcare workers were aware of MVD.^19^ The heavy reliance on online resources for information raises concerns about misinformation, as highlighted during the Ebola outbreak in Uganda where over 70% of healthcare workers cited social media as their primary information source.^20^

The minimal formal training rates (19.1% of doctors, 10.0% of nurses) represent a critical vulnerability in Nigeria’s outbreak preparedness. This deficit is particularly concerning given that healthcare workers would serve as first responders during any MVD outbreak. Similar training inadequacies have been documented in other viral haemorrhagic fever contexts,^21,22^ suggesting systemic weaknesses in preparedness education across the region.

The paradoxical finding that nurses expressed higher confidence in hospital preparedness than doctors (65.6% vs. 32.5%) warrants careful interpretation. This disparity may reflect nurses’ closer involvement in routine patient care protocols and greater familiarity with existing infection control measures. Conversely, doctors’ lower confidence might stem from a deeper understanding of the technical and logistical complexities required for MVD case management, including sophisticated laboratory diagnostics and intensive care capabilities.

The finding that less than half of healthcare workers felt adequately equipped with PPE represents a critical preparedness gap. This inadequacy mirrors challenges observed during previous viral haemorrhagic fever outbreaks^19^ and could significantly compromise healthcare worker safety and confidence during an actual outbreak. The absence of established protocols in many departments (63.5% of doctors reporting no protocols) further undermines institutional preparedness.

Although this study did not directly assess infection prevention and control (IPC) knowledge, similar research across Nigeria has consistently reported gaps in healthcare workers’ adherence to standard precautions and infection control practices.^23,24,25,26,27,28^ These contextual findings underscore the likelihood that such gaps may also influence preparedness for MVD and other viral haemorrhagic fevers. Consequently, strengthening training programmes that integrate IPC principles with outbreak-specific preparedness remains essential.

The coronavirus disease 2019 (COVID-19) pandemic demonstrated the transformative potential of digital technologies in outbreak response, from telemedicine platforms to artificial intelligence-powered surveillance systems.^29,30^ These lessons offer valuable opportunities for addressing the identified preparedness gaps. Technology-enhanced training programmes could standardise MVD education across professional groups, while mobile health applications could facilitate real-time protocol updates and competency assessments.^31,32^ Virtual reality simulation training has shown promise in enhancing emergency response capabilities among healthcare workers.^33^ Digital surveillance platforms could also improve PPE inventory management and supply chain coordination.^34,35^

The importance of systematic training and education programmes for viral epidemic response has been emphasised in recent literature.^36^ Such programmes should incorporate both traditional and innovative approaches to ensure comprehensive preparedness among healthcare workers. The experience from previous outbreaks, including Ebola, has demonstrated that well-trained and prepared healthcare workers are essential for effective outbreak response and containment.^37,38^

### Recommendations

Based on these findings, several critical interventions are needed to strengthen MVD preparedness in Nigeria:

1. *Training and Education:* Implement comprehensive, role-specific MVD training programmes utilising both traditional and digital platforms. Regular workshops, simulation exercises and online modules should address knowledge gaps, particularly among nursing staff. Training should encompass symptom recognition, transmission pathways, infection control measures and case management protocols.
2. *Infrastructure Development:* Ensure adequate PPE stockpiles across all departments with regular inventory assessments. Establish clear, evidence-based protocols for MVD case management and ensure all staff are familiar with these procedures. Improve diagnostic capabilities and ensure rapid laboratory confirmation processes are in place.
3. *Digital Integration:* Leverage digital health technologies to enhance training delivery, competency assessment and real-time protocol updates. Implement digital surveillance systems for outbreak monitoring and resource allocation. Utilise mobile health platforms for continuous education and emergency communication.
4. *Interdisciplinary Collaboration:* Foster collaborative training sessions between doctors and nurses to ensure cohesive outbreak response. Establish multidisciplinary rapid response teams with clearly defined roles and responsibilities.
5. *Continuous Monitoring:* Implement regular assessments of healthcare worker preparedness and knowledge levels. Establish feedback mechanisms to continuously improve training programmes and preparedness protocols.

### Strengths and limitations

This study’s primary strength lies in its focus on Nigeria’s principal viral haemorrhagic disease reference centre, providing insights directly relevant to the country’s outbreak response capacity. The substantial sample size (216 participants) and inclusion of both doctors and nurses enabled meaningful comparative analysis of professional preparedness differences. The comprehensive assessment covering knowledge, attitudes and preparedness provided multidimensional insights into healthcare worker readiness.

However, several limitations must be acknowledged. The single-centre design limits generalisability to other Nigerian healthcare facilities, particularly those without specialised viral haemorrhagic disease expertise. The study relied on self-reported perceptions and knowledge rather than objective competency assessments, which may introduce response bias. The cross-sectional design prevents assessment of knowledge and preparedness changes over time. Additionally, the study did not examine broader institutional factors such as hospital infrastructure, funding constraints or policy frameworks that influence preparedness capacity.

Furthermore, the study did not assess participants’ prior training or experience in managing other viral haemorrhagic fevers. Although healthcare workers at the study site routinely receive training related to Lassa fever management, most have limited or no formal exposure to MVD or other haemorrhagic fevers. This represents a potential limitation, as preparedness and disease-specific knowledge may differ between Lassa fever and other viral haemorrhagic infections. Future research should address these limitations through multicentre studies, objective competency assessments and broader institutional analysis.

## Conclusion

This study reveals substantial gaps in MVD health literacy and outbreak preparedness among healthcare workers at Nigeria’s primary viral haemorrhagic disease reference centre. Professional disparities in knowledge, minimal formal training opportunities and inadequate protective equipment represent critical vulnerabilities in Nigeria’s outbreak response capacity. The paradoxical confidence levels between doctors and nurses highlight the complexity of preparedness assessment and the need for nuanced interventions.

Urgent action is required to implement comprehensive training programmes, strengthen preparedness infrastructure and leverage digital health technologies to enhance outbreak readiness. By addressing these gaps, Nigeria can significantly improve its capacity to respond effectively to MVD outbreaks and protect both healthcare workers and the broader population. These findings also raise important questions about existing protocols at viral haemorrhagic fever reference centres and the resources required to mount effective outbreak responses in Nigeria.

The COVID-19 pandemic demonstrated that preparedness investments made before outbreaks occur yield far greater returns than reactive responses. Nigeria must learn from these experiences and invest in healthcare worker preparedness now, before the next viral haemorrhagic fever outbreak occurs.
